# Fallbericht zur Entstehung einer medialen Gonarthrose nach Innenmeniskusläsion und erfolgter Teilresektion

**DOI:** 10.1007/s00113-022-01173-0

**Published:** 2022-04-13

**Authors:** Felix Finger, Marc-Daniel Ahrend, Christoph Ihle, Tina Histing, Steffen Schröter

**Affiliations:** 1 BG Unfallklinik Tübingen, Klinik für Unfall- und Wiederherstellungschirurgie, Eberhard Karls Universität Tübingen, Schnarrenbergstr. 95, 72076 Tübingen, Deutschland; 2 Klinik für Unfall- und Wiederherstellungschirurgie, Diakonie Klinikum Jung-Stillig GmbH, Siegen, Deutschland

**Keywords:** Meniskusläsion, Meniskusresektion, Knorpeldegeneration, Kniearthrose, MRT, Meniscus lesion, Meniscectomy, Cartilage degeneration, Gonarthrosis, MRI

## Abstract

Meniskusverletzungen führen zur Veränderung der Belastungsverteilung im Kniegelenk. Das Risiko, eine Gonarthrose zu entwickeln, steigt mit Zunahme der resezierten Meniskusfläche. Der Fallbericht zeigt, basierend auf 4 MRT-Untersuchungen, die über einen Zeitraum von 8 Jahren stattfanden, die fortschreitende Knorpeldegeneration nach traumatischer Innenmeniskusläsion und resultierender Teilresektion eines zum Unfallzeitpunkt 46-jährigen Patienten. Angeborene oder unfallunabhängige Risikofaktoren wie eine varische Beinachse müssen bei einer möglichen Begutachtung Berücksichtigung finden.

## Abstract (en)

Meniscal injuries lead to changes in pressure distribution in the knee joint. The risk of developing gonarthrosis increases by the amount of resected meniscal tissue. Based on 4 MRI examinations conducted over an 8‑year time period, the present case report shows the progress of cartilage degeneration after a traumatic medial meniscus lesion and subsequent partial meniscus resection in a 46-year-old male patient. Congenital or accident-independent risk factors, such as a varus limb deformity must be considered during a medical report.

## Hintergrund

Der häufigste Grund für arthroskopische Eingriffe am Kniegelenk sind Operationen am Meniskus. Das Ausmaß der Resektion bei Meniskektomien, Meniskusnaht oder -resektion wird kontrovers diskutiert [20]. Ein Konsens besteht jedoch für den Zusammenhang zwischen fortschreitendem Verlust an Meniskusfläche und Risiko für die Entstehung einer Gelenkarthrose [10, 22]. Konsekutive MRT-Untersuchungen, die den Verlauf einer fortschreitenden Reduktion an Meniskusfläche in kurzen Zeitabständen porträtieren, liegen selten vor. Die vorliegende Falldarstellung umfasst, über einen Zeitraum von 8 Jahren, 4 MRT-Untersuchungen. Eine Kausalitätsprüfung bezüglich der Ätiologie der Gonarthrose muss diverse Faktoren berücksichtigen. Die engmaschige Verfolgung des Krankheitsverlaufes mittels MRT-Sequenzen kann hierzu einen wertvollen Beitrag leisten und wird anhand des folgenden Fallberichtes dargestellt.

## Fallbericht

### Anamnese

Ein zum Unfallzeitpunkt 46-jähriger, männlicher Patient (BMI 25 kg/m^2^) stellte sich im Juli 2011 mit einem geschwollenen rechten Kniegelenk nach Ausrutschen und Anpralltrauma ohne bekannten Vorschaden extern vor. Vor dem Unfall war der Patient beschwerdefrei. Unfallunabhängig bestand eine posttraumatische Koxarthrose links, bedingt durch eine Acetabulumfraktur vor über 30 Jahren. Eine Hüfttotalendoprothese wurde im September 2017 implantiert.

### Therapie und Verlauf

**Figure 1:**
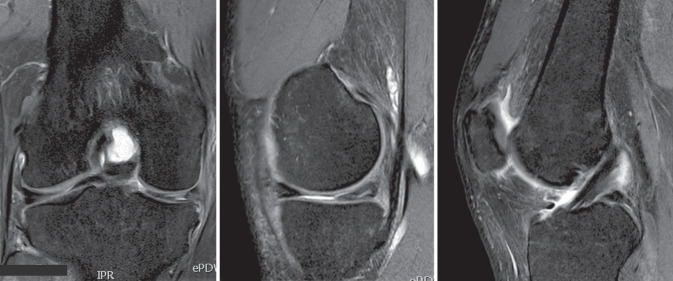

Die erste MRT-Untersuchung im September 2011 (Abb. 1) zeigt eine Innenmeniskus(IM)-Läsion in Form eines Horizontalrisses des Hinterhorns (HH), bis in die Pars intermedia und an die Meniskusbasis reichend (Cooper-Zone 1) [9]. Der Knorpelüberzug im medialen sowie im lateralen Kompartiment zeigt keine Pathologie. Das vordere Kreuzband (VKB) war durchgängig darstellbar, allenfalls im Sinne einer Partialruptur signalalteriert. Zur Meniskussanierung wurde eine Arthroskopie des Kniegelenks durchgeführt. Der intraoperative Befund wurde als traumatisch bedingte IM-HH-Läsion mit I.°-Knorpelläsion (Outerbridge) dokumentiert [18, 19]. Intraoperativ zeigten sich stabile Bandverhältnisse mit adäquater Lastaufnahme des VKB. Therapeutisch wurden, bei komplexem IM-Riss, bis in die Basis reichend (Cooper-Zone 1), partielle Teilresektionen des IM-HH und der Pars intermedia durchgeführt.

Bei persistierenden Beschwerden wurde eineinhalb Jahre später (Februar 2013) ein weiteres MRT durchgeführt, welches einen ausgedehnten Substanzdefekt des IM nach Teilresektion mit degenerativen Veränderungen im HH-Bereich zeigte. Zudem zeigte sich bereits eine III.°-Knorpelschädigung (Outerbridge) mit Verschmälerung des Knorpelbelags und resultierendem Knochenmarködem femoral und tibial im medialen Kompartiment. Bei Beschwerdepersistenz erfolgte extern eine erneute Arthroskopie mit diagnostischem Zweck. Neben einem stabilen, subtotal resezierten IM, stellte sich ein III.- bis IV.°-Knorpelschaden (Outerbridge) an der Femurkondyle und am Tibiaplateau dar.

**Figure 2:**
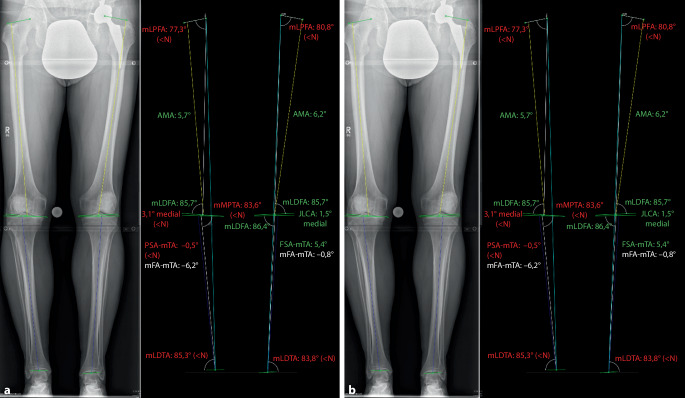

Bei der erstmaligen Vorstellung in der BG Unfallklinik Tübingen zeigte sich in der klinischen Untersuchung im April 2014 eine Streckhemmung von 5° bei stabilen Seitenbandverhältnissen und hartem Anschlag des VKB im Lachmann-Test. Die durchgeführte Röntgendiagnostik des Kniegelenks stellt eine diskrete mediale Gelenkspaltverschmälerung dar. Auf der Ganzbeinstandaufnahme zeigt sich eine varische Beinachse mit einem mechanischem tibiofemoralen Winkel (mTFA) von −3° bei einem mechanischem medialen proximalen Tibiawinkel (mMPTA) von 86°, einem mechanischem lateralen distalen Femurwinkel (mLDFA) von 87,4° und einem joint line convergence angle (JLCA) von 1,6° rechts. Auf der linken Seite war die Beinachse mit einem mTFA von 0,7° gerade (Abb. 2).

**Figure 3:**
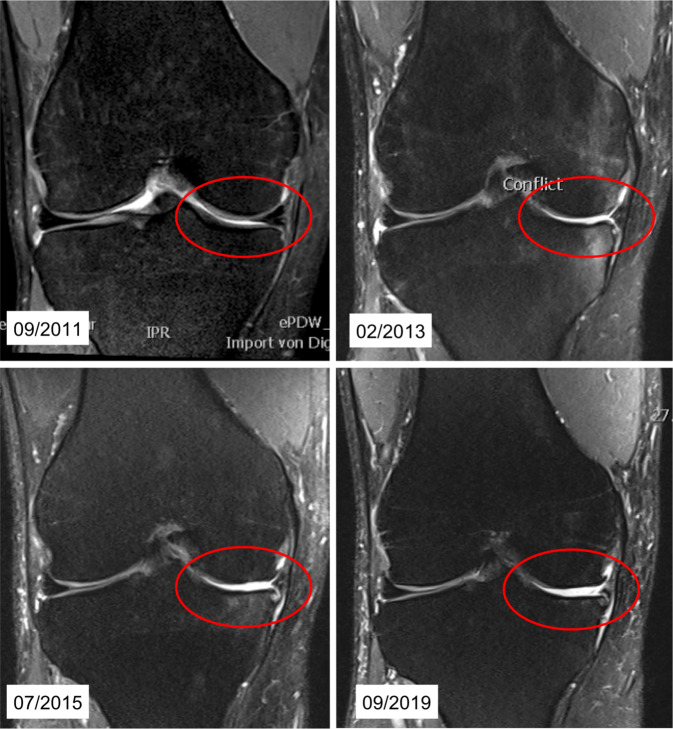

Aufgrund der klinischen und radiologischen Befunde wurde dem Patienten die Durchführung einer Arthroskopie und einer valgisierenden hohen Tibiakopfumstellungsosteotomie (HTO), mit dem Ziel der Entlastung des medialen Kompartiments, sowie ggf. zum Ausgleich der Streckhemmung die Anhebung des tibialen Slope geraten. Der Patient lehnte ein operatives Vorgehen ab. Im Verlauf wurde bei persistierenden medialen Kniebeschwerden ein weiteres MRT (Juli 2015) durchgeführt. Dieses ergab einen ausgiebigen IM-Defekt mit Einrissen am IM-HH mit Progression der Fläche des medialen Knorpelschadens sowie einhergehendem tibialen und femoralen Knochenmarködem und Baker-Zyste. Der Patient ließ extern eine weitere Arthroskopie durchführen. Hier wurde eine Nachresektion eines Rezidiveinrisses am IM durchgeführt. Erneut wurde dem Patienten eine HTO bei Beschwerdepersistenz empfohlen. Aufgrund eines ausbleibenden Therapieerfolgs wurde nach 4 weiteren Jahren ein 4. MRT (September 2019) durchgeführt, das eine weiter fortschreitende, medial betonte Gonarthrose zeigt. Das laterale Kompartiment zeigt keine Beeinträchtigung der Knorpelschicht. Die 4 MRT Untersuchungen sind in Abb. 3 zusammengefasst.

Acht Jahre nach dem Unfallereignis stellt sich der Patient zur Begutachtung in der BG Unfallklinik Tübingen vor. Es zeigt sich ein diskret rechts hinkendes Gangbild mit einem limitierten Bewegungsumfang der Streckung/Beugung 0‑10-130°. Das VKB zeigt eine diskret verlängerte Translationsstrecke bei festem Anschlag. Der Patient klagt weiterhin über belastungsabhängige Schmerzen.

## Diskussion

Anhand der chronologischen MRT-Untersuchungen kann eine stetige Knorpeldegeneration im medialen Kompartiment nach IM-Läsion und subtotaler Resektion demonstriert werden. Es zeigte sich auch eine Zunahme der Ausprägung der varischen Beinachse. Eine Dokumentation der Beinachse auf Ganzbeinstandaufnahmen zum Unfallzeitpunkt lag nicht vor. Drei Jahre posttraumatisch zeigte sich eine varische Beinachse (mFTA: −3°) rechts, wohingegen die unverletzte Seite keine Achsabweichung zeigte. Acht Jahre nach dem Unfall stellte sich eine mediale Gonarthrose bei zunehmend varischer Beinachse (mFTA: −6°) dar (Abb. 2).

Meniskusschäden und deren Einfluss auf die Entstehung der Kniearthrose sind Gegenstand vieler Untersuchungen. Englund et al. diskutieren beispielsweise die Rolle der Meniskusläsion im Zusammenhang mit Kniearthrose als Ursache oder Folge [11]. Eine pauschale Aussage hierüber konnte nicht getroffen werden. Es ist sowohl möglich, dass ein Meniskusriss die Arthrose verursacht, als auch, dass die Entstehung einer Meniskusläsion auf Basis einer vorbestehenden Arthrose gründet [11]. Eine individuelle Prüfung zur Zusammenhangsbeurteilung ist dementsprechend wichtig. Im vorliegenden Fall zeigte sich durch die IM-Teilresektion und mutmaßliche Durchtrennung der zirkumferenten Fasern bereits im ersten Folge-MRT ein Knorpelschaden. Ferner ist in der koronaren Schicht bereits keine Meniskussubstanz mehr nachweisbar. In der Kausalitätsargumentation sind diverse Faktoren zu berücksichtigen, die als interagierendes Konstrukt evaluiert und beispielsweise im Rahmen einer Begutachtung beurteilt werden müssen.

Meniskusläsionen werden in primäre, traumatisch-bedingte und sekundäre, degenerative Meniskopathien unterschieden. Letztere treten zunehmend ab dem 40. Lebensjahr auf. Wiederkehrende, kniegelenkbelastende Tätigkeiten und harte Beanspruchungen mit ungünstigen Gelenkstellungen können chronische Meniskusschäden verursachen [17]. Im Gegensatz hierzu stehen die traumatischen Meniskusverletzungen, die einen bestimmten Unfallmechanismus voraussetzen [14]. Als geeignete Unfallmechanismen für einen isolierten Meniskusriss gelten eine gewaltsame Verdrehung des Unterschenkels gegenüber dem Oberschenkel oder ein Beuge-Dreh-Sturz, bei dem der Fuß fixiert ist. Hierbei treten häufiger longitudinale Risse oder Radiärrisse auf [14]. Isolierte Horizontalrisse des Meniskus sind häufiger degenerativer Ursache und sind arthroseassoziiert. Patienten > 50 Jahre, mit BMI > 25 kg/m^2^, weiblichem Geschlecht oder einer Varusanlage haben eine erhöhte Wahrscheinlichkeit, einen medialen HH-Riss zu erleiden, im Vergleich zu anderen Meniskusläsionen [13]. Jedoch können auch jüngere Patienten isolierte Horizontalrisse erleiden. Terzidis et al. [26] evaluierten 378 isolierte Meniskusverletzungen in jungen Athleten und berichteten über 17,4 % Horizontalrisse, signifikant häufiger am medialen Meniskus, im Vergleich zum lateralen Meniskus. Wie von Kim et al. beschrieben, erstreckt sich die Läsion bei traumatisch bedingten, isolierten Horizontalrissen weiter, meist über das HH und Pars intermedia, im Vergleich zu degenerativen Horizontalrissen [14]. In vorliegendem Fall war der Patient männlich, 46 Jahre, normgewichtig, das Kniegelenk zeigte einen intakten Knorpelüberzug, und der Riss streckte sich vom HH bis in die Pars intermedia. Obwohl die Genese von Horizontalrissen in den meisten Fällen degenerativ ist, muss im vorliegenden Fall im Hinblick auf die Kausalitätsprüfung das adäquate Trauma mit Bedacht werden. Sollte eine asymptomatische, degenerative Veränderung vorbestanden haben, führte trotzdem das Trauma zu akuten Beschwerden und der resultierenden Arthroskopie mit Teilresektion.

Die biomechanische Folge einer Meniskusläsion entsteht durch Verletzungen der zirkumferenten Fasern, welche v. a. bei radiärer Rissbildung oder bei Entfernung von Teilen des Meniskus entstehen. Somit resultieren eine Reduktion der möglichen Ringspannung und die Reduktion der Kontaktfläche mit Extrusion des Meniskus. Es folgen ein Anstieg des Kontaktdruckes und somit die Erhöhung des Risikos für eine Arthrose [3]. Seitz et al. konnten in ihrer biomechanischen Kadaverstudie zeigen, dass eine vollständige Teilresektion auf 10 mm Breite im Bereich des medialen Hinterhorns je nach Flexionsgrad zu einer 47- bis 68 %igen Erhöhung des maximalen Kontaktdrucks, im Vergleich zum intakten Zustand, führt. Eine partielle Teilresektion des medialen Meniskus bis zu 20 % Tiefe und bis 10 mm Breite führte jedoch zu keiner Erhöhung des tibiofemoralen Kontaktdrucks im medialen Gelenkskompartiment [24]. Marzo et al. führten eine komplette Durchtrennung des medialen Meniskus nahe des Hinterhorns durch und konnten eine Steigerung des maximalen Kontaktdrucks im medialen Kompartiment von 3841 kPa auf 5084 kPa (*p* = 0,006) und eine Reduktion der tibiofemoralen Kontaktfläche von 594 mm^2^ auf 474 mm^2^ (*p* = 0,005) feststellen [15]. Folglich ist die Erhaltung der Meniskusbasis in jedem Fall anzustreben.

Im vorliegenden Fall zeigte sich eine zunehmende, medial betonte Gelenkspaltverschmälerung mit Knorpelreduktion und Zunahme der varischen Beinachse. Aufgrund der fehlenden Ganzbeinstandaufnahme konnte die mechanische Beinachse zum Unfallzeitpunkt nicht eruiert werden. Auf der Gegenseite zeigte sich eine physiologische Beinachse in den im Behandlungsverlauf durchgeführten Aufnahmen. Colyn et al. zeigten an 54 Fußballspielern keinen signifikanten Unterschied des mittleren mTFA zwischen dem dominanten (−2,6 ± 2,2) und nichtdominanten Bein (−3,0 ± 2,5) [8]. Es erscheint plausibel, dass in vorliegendem Fall am rechten Bein zum Unfallzeitpunkt eine physiologische Beinachse vorgelegen hat. Die mechanische Beinachse bei 250 gesunden Männern betrug im Mittel (± SD) −1,87° ± 2,42° varus, und bei 32 % der gesunden Männer fand sich ein konstitutioneller Varus von −3° oder weniger [1].

Eine Zunahme einer varischen Beinachse durch eine mediale Meniskusresektion konnte durch Yoon et al. gezeigt werden. Die Beinachsenveränderung wurde bei 56 Patienten mit partieller oder totaler Meniskektomie über einen Zeitraum von durchschnittlich 6,7 Jahren gemessen. Vor der Meniskektomie zeigte das Kollektiv einen Varus von 2,4° ± 2,4°. Eine Zunahme des Varus von durchschnittlich 1,7° ± 1,5° (Minimum: 0, Maximum: 6,9°) wurde beobachtet. Das Ausmaß der Veränderung war hierbei positiv korrelierend mit der Resektionsfläche [29]. Dies deckt sich mit den Beobachtungen im vorliegenden Fall. Bezüglich der Kausalitätsprüfung kann festgehalten werden, dass bei einer allenfalls konstitutionellen Varusanlage im physiologischen Bereich die verstärkte Varusabweichung auf den Substanzverlust des IM zurückzuführen ist.

Der Einfluss einer varischen Beinachse auf die Entstehung einer medialen Gonarthrose ist nicht abschließend geklärt [27]. Sharma et al. und Brouwer et al. beschrieben ein Varus-Alignment als Risikofaktor für die Entstehung einer medialen Gonarthrose [5, 25], während Hunter et al. keinen Zusammenhang hierfür finden konnten [12]. Bezüglich des Einflusses einer varischen Beinachse auf das Fortschreiten einer bestehenden Arthrose zeigen Studien eine höhere Wahrscheinlichkeit der Arthroseprogression bei Vorliegen einer varischen Beinachse gegenüber Kontrollen mit neutraler Beinachse ((OR 2,90; 95 %-KI 1,07–7,88 [5]), (OR 4,12; 95 %-KI 1,92–8,82 [6]), (OR 3,59; 95 %-KI 2,62–4,92 [25])). Darüber hinaus zeigen biomechanische Untersuchungen von Willinger et al., dass der Kontaktdruck im medialen Kniegelenkkompartiment nach einer Teilresektion des medialen Meniskus insbesondere bei varischer Beinachse zunimmt [28]. Hieraus ergibt sich eine ungünstige Dynamik bezüglich der fortschreitenden Arthrose.

Vor dem Hintergrund der eben genannten Erkenntnisse muss die operative Versorgung mittels Teilresektion, wie sie in diesem Fallbeispiel durchgeführt wurde, kritisiert werden. Chung et al. verglichen in ihrer Studie Teilresektion und Wiederfixierung von IM-HH-Läsionen und konnten sowohl klinische als auch radiologische Vorteile der Wiederfixierung zeigen [7]. Die Follow-up-Zeit betrug mindestens 5 Jahre, und insbesondere die Werte des Lysholm-Scores und der Arthroseprogression anhand der Kellgren-Lawrence-Skala zeigten eine Überlegenheit der Wiederfixierung. Ebenso konnten Bernard et al. Vorteile einer Wiederfixierung bei IM-HH-Rissen gegenüber der Teilresektion und der konservativen Therapie zeigen [2]. Auch wenn die Tendenz zur Überlegenheit der operativen Wiederfixierung von IM-HH-Rissen geht, resümieren die Autoren, dass weitere Studien zur Definierung der optimalen Behandlung notwendig sind [2, 7]. Es sind auch Konzepte vorhanden, die eine kombinierte Versorgung mittels Teilresektion und Naht vorsehen, um den Anteil an zu erhaltendem Meniskusgewebe zu maximieren. Bei jungen Patienten konnten hier gute Ergebnisse erzielt werden [4, 21]. Ob sich im aktuellen Fall mit einer anderen Versorgung ein Vorteil für den Patienten ergeben hätte, lässt sich abschließend nur vermuten.

Begleitende Pathologien von HH-Läsionen beeinflussen das Beschwerdebild. Der VKB-Zustand im vorliegenden Fall wurde im ersten MRT als fragliche Partialruptur beschrieben. Nach Kniedistorsion tritt eine VKB-Ruptur eher bei Außenmeniskus-HH-Rupturen als bei IM-HH-Läsion auf, wie Matheny et al. zeigten konnten. Demnach besteht eine 10,3-fach erhöhte Wahrscheinlichkeit, eine VKB-Ruptur bei Außenmeniskus-HH-Riss zu haben, gegenüber Fällen mit vorliegender IM-HH-Läsion [16]. Es ist bekannt, dass Patienten mit einer VKB-Läsion ein erhöhtes Risiko für Gonarthrose und sekundäre Rissbildungen am Meniskus haben [23]. Im präsentierten Fall zeigte sich das VKB stets mit festem Anschlag in der klinischen Untersuchung, sodass die MR-tomographisch beschriebene Partialläsion für die Kausalitätsprüfung wohl keinen relevanten Einfluss auf das Gesamtgeschehen der Arthroseentwicklung hatte.

## Fazit für die Praxis

Anhand der dargestellten chronologischen MRT-Untersuchungsreihe wird gezeigt, dass die IM-Läsion mit resultierender subtotaler Meniskusresektion ursächlich für die sukzessive Knorpeldegeneration im medialen Gelenkkompartiment ist. In einer allfälligen Begutachtung kann somit die Aussage gestützt werden, dass daraus im Verlauf eine manifeste, mediale Gonarthrose mit varischer Achsabweichung resultierte.
